# Rapamycin nanoparticles improves drug bioavailability in PLAM treatment by interstitial injection

**DOI:** 10.1186/s13023-022-02511-6

**Published:** 2022-09-09

**Authors:** Yahong Shi, Chuqiao Jiao, Xi Lu, Yifeng Nie, Xiang Li, Dong Han

**Affiliations:** 1 School of Life Sciences, Beijing University of Chinese Medicine, Beijing, 100029 China; 2 National Center for Nanoscience and Technology, Beijing, 100190 China; 3 Beijing City International School, Beijing, 100022 China

**Keywords:** Pulmonary lymphangiomyomatosis (PLAM), Rapamycin, Intervaginal space injection (ISI), LC–MS

## Abstract

**Background:**

Pulmonary lymphangiomyomatosis (PLAM) is a rare interstitial lung disease characterized by diffuse cystic changes caused by the destructive proliferation of smooth muscle-like cells or LAM cells. PLAM is more common in young women than other people, and a consensus is lacking regarding PLAM treatment. The clinical treatment of PLAM is currently dominated by rapamycin. By inhibiting the mTOR signaling pathway, rapamycin can inhibit and delay PLAM’s occurrence and development. However, the application of rapamycin also has shortcomings, including the drug’s low oral bioavailability and a high binding rate to hemoglobin, thus significantly decreasing the amount of drug distributed to the lungs.

**Methods and results:**

Here, we developed a new mode of rapamycin administration in which the drug was injected into the intrathecal space after being nanosized; the directional flow characteristics of the liquid in the intrathecal space were exploited to increase the drug content in the interstitial fluid to the greatest extent possible. We studied the rapamycin content in the interstitial fluid and blood after intervaginal space injection (ISI). Compared with oral administration, ISI significantly increased the drug concentration in the lung interstitial fluid.

**Conclusions:**

These results provided new ideas for treating PLAM and optimizing the dosing regimens of drugs with similar characteristics to rapamycin.

## Background

**Fig. 1:**
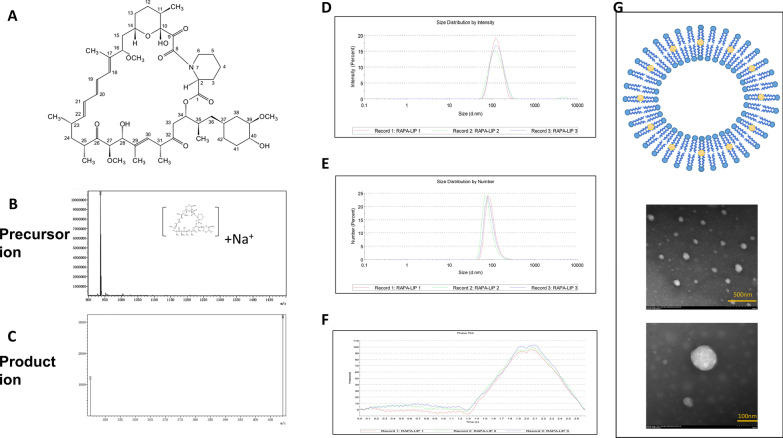
Ion mass spectras of rapamycin and parameters of rapamycin liposomes. **A** Chemical structure of rapamycin. **B** Precursor ion mass spectra (m/z 936) of rapamycin. **C** Product ion mass spectras (m/z 409 and m/z 345) of rapamycin. **D** Particle size distribution of rapamycin liposomes by intensity. **E** Particle size distribution of rapamycin liposomes by number. **F** Zeta potential phase plot of rapamycin liposomes. **G** Schematic diagram and transmission electron microscopy images of the rapamycin liposomes. Scale bars, 500 nm and 100 nm

Pulmonary lymphangiomyomatosis (PLAM) is a rare lung disease belonging to the perivascular epithelioid cell family of tumors. PLAM is characterized by diffuse cystic changes caused by destructive proliferation of smooth muscle-like cells or LAM cells. PLAM is more common in young women than other people, and a consensus regarding PLAM treatment is lacking. Rapamycin, a macrocyclic lactone, is a potent immunosuppressive agent (Fig. 1A). It is clinically used to treat PLAM and immune rejection after organ transplantation. Treatment with rapamycin is strongly recommended over other treatments by clinicians, because of its clear effects [1]. Patients with LAM have TSC gene inactivation mutations [2], which ultimately lead to the disruption of cell development, motility and survival through activation of the mechanistic target of rapamycin (mTOR) signaling pathway. Rapamycin restores the homeostasis of TSC-deficient cells by inhibiting the mTOR pathway. However, the clinical application of rapamycin has several shortcomings. First, the bioavailability of oral rapamycin is low [3]; second, because rapamycin specifically binds hemoglobin, 95% of the drug is sequestered in red blood cells [4], thus diminishing the effects of the drug. To solve these problems, in this study, we prepared rapamycin liposomes and used a new method of intervaginal space injection (ISI). The concentration of rapamycin in the whole blood and pulmonary interstitial fluid was measured with liquid chromatography-mass spectrometry (LC–MS). Compared with the traditional mode of rapamycin administration, the administration of rapamycin liposomes through ISI effectively increased the drug content and bioavailability in the lung tissue interstitium and decreased drug clearance. Our results provided new ideas for clinical medication to regulate the steady state concentration of rapamycin.

## Materials and methods

### Animals

A total of 48 healthy female C57/BL6N mice (weighing 20 ± 2 g) were obtained from Beijing Vitong Lihua Co., LTD. The mice were kept in a regulated environment (12-h dark/light cycle, 22 ± 2 °C temperature and 50 ± 5% humidity).

### Drugs and chemicals

Rapamycin (CAS no. 53123-88-9, purity > 98%), soya bean lecithin and methyl tert-butyl ether (MTBE, HPLC-grade) were provided by Macklin Inc. (Shanghai, China). Cholesterol was purchased from Solarbio Life Sciences Inc. (Beijing, China). Methanol (HPLC-grade) and formic acid were purchased from Thermo Fisher Scientific Inc. (Pittsburgh, PA, USA).

### Methods

#### Preparation of rapamycin liposomes

Rapamycin liposomes were prepared through the thin film dispersion method. First, soybean lecithin, cholesterol and rapamycin (molar ratio of 100:25:4) were dissolved in an appropriate amount of anhydrous ethanol. After 15 min of ultrasound treatment in a water bath, the materials and drug were completely dissolved. The ethanol was evaporated with a rotary evaporator at 40 °C. Then 10 mL of normal saline was added, and hydration proceeded at 55 °C for 30 min. After ultrasound treatment in a water bath for 15 min, a liposome mini-extruder (AVANTI POLAR LIPIDS INC., Alabama, USA) was used to extrude liposomes 100 nm in diameter, and the operation was repeated 20 times. Centrifugation at 3000 × g for 10 min was performed to remove the unencapsulated drug.

After dilution of the prepared rapamycin liposomes with an appropriate amount of water, the hydrated particle size and zeta potential were measured with a size analyzer (Zetasizer Nano ZS, Malvern, UK).

Subsequently, 10 µL of diluted rapamycin liposomes was deposited on copper mesh and allowed to stand for 10 min. The upper layer solution was then aspirated, and 10 µL of 2% uranyl acetate was added for negative staining. After 8 min, the upper layer solution was aspirated. The mesh was washed with double distilled water, allowed to dry and observed with a transmission electron microscope (HT7700, Hitachi, Japan).

#### Instrumentation and chromatographic conditions

Analyte separation was achieved with a Shim-pack GIST-HP C18 analytical column (2.1 × 100 mm, 3.0 µm). Gradient elution was performed with phase A of 0.1% formic acid and phase B of methanol, with a starting ratio of 17:83 (v/v) and a flow rate of 0.2 mL/min. The gradient elution run time was 6 min, and the injected sample volume was 2 μL.

Detection was performed on a Shimadzu 8040 LC–MS/MS instrument equipped with an electrospray ionization source. Rapamycin was determined in positive ion mode. The optimal parameters were set as follows: reaction monitoring was selected with transitions of m/z 936-409 for rapamycin (Fig. 1B, C); the collision energy value was set to 55 mV for rapamycin.

#### Preparation of standards and quality control standard solution

A rapamycin stock solution (10 mg/mL) was prepared with methanol. Working solutions of rapamycin and calibration curves were generated by serial dilution of the stock solution with methanol (0.001 µg/mL, 0.005 µg/mL, 0.01 µg/mL, 0.05 µg/mL, 0.1 µg/mL, 0.15 µg/mL, 0.5 µg/mL, and 1 µg/mL). Low-, mid- and high- level quality control samples were prepared by spiking standard solutions into biological samples (0.5, 0.1, and 0.02 μg/mL for whole blood and lung tissue). Working solutions and calibration standards were prepared immediately before use.

#### Sample preparation

**Fig. 2:**
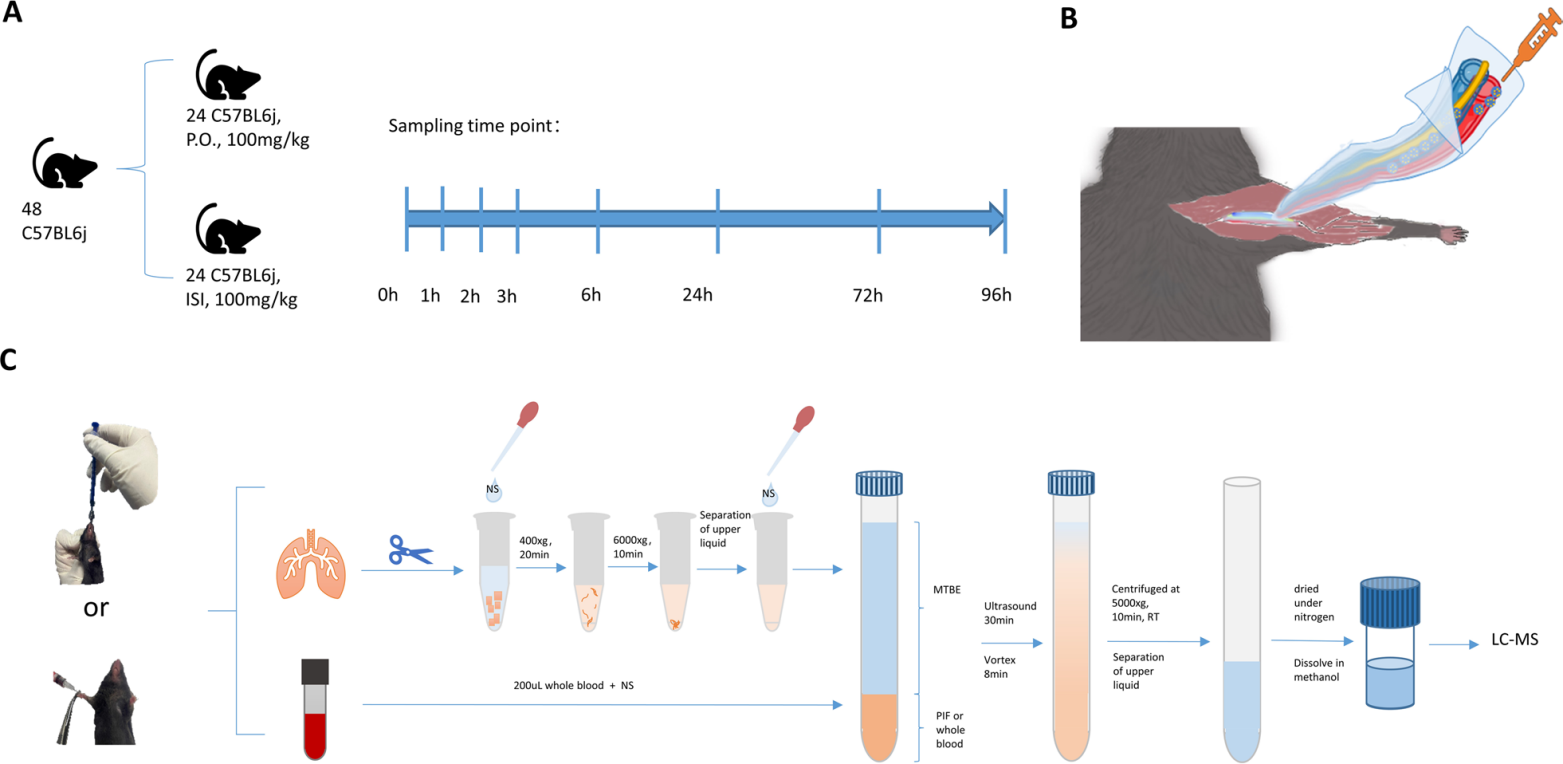
Pharmacokinetic experimental design. **A** Grouping arrangement and sampling time points of this experiment. **B** Schematic diagram of the intervaginal space injection (ISI) injection site. **C** Sample processing flow chart

The rapamycin in biological samples was concentrated by liquid–liquid extraction for detection. The detailed steps are described below. For blood samples, 200 µL of anticoagulated whole blood was collected. Then 1 mL of normal saline was added to dilute the sample, and 3 mL of methyl tert-butyl ether was added for extraction. The mixture was vortexed for 30 s, ultrasonicated for 30 min in a water bath, vortexed again for 8 min and centrifuged at 5000 × *g* for 10 min at RT. The upper ether solution was collected and dried under nitrogen. Then the residue was dissolved in 800 µL methanol. Samples were then ready for analysis. For tissue samples, the tissue was weighed, 500 µL of normal saline was added, and the tissue was minced. The interstitial fluid was extracted according to a previously described method [5]. The subsequent procedures were as described above. Sample processing flow chart is shown in Fig. 2C.

#### Pharmacokinetic study

Mice were randomly divided into two groups: an oral (P.O.) group and an ISI group. The specific administration of interstitial injection was as previously described [6] (Fig. 2B). In simple terms, the injection site is located at the carpal tunnel of the wrist bone, between the flexor carpal ulnar and flexor carpi radialis. It is a convergent point for tendons, vessels, and nerve fibers connected to the fascia surrounding them. The operator tilts the syringe into the injection site. A pronounced feeling of emptiness can be felt after the piercing, there is no obstruction in nudging the syringe, and there is no return of blood. The dose of rapamycin was 100 mg/kg in both groups. Eight time points were sampled (0, 1, 2, 3, 6, 24, 72, and 96 h) with three mice each (Fig. 2A). The eyeballs of the mice were removed for blood collection after anesthesia with isoflurane. Lung tissue was removed after cardiac perfusion with normal saline to remove blood in the lungs. All samples were stored at − 20 °C until analysis.

### Statistical analysis

The concentrations in the samples were determined on the basis of calibration curves, and drug-time curves were plotted. Pharmacokinetic parameters were calculated according to the drug-time curves, including time to peak (T_max_), maximum capacity (C_max_), area under the curve at 72 h (AUC_0→72 h_), clearance (CL) and multiple of bioavailability (F).

## Results

### Particle size, zeta potential and morphology of rapamycin liposomes

The particle size and zeta potential of the nanoparticles are shown in Table 1 and Fig. 1D–F. The rapamycin liposomes had a desirable average particle size of 120.9 nm and a narrow size distribution. The encapsulation efficiency of liposomes, as detected by ultraviolet absorption spectroscopy, was 98.20 ± 0.15%. Almost all the drug was packed in liposomes, with little remainder.

**Table 1:** Particle size and zeta potential of rapamycin liposome (n = 3)

Formulation	Particle size (nm)	Polydispersity index	Zeta potential (mV)	Encapsulation efficiency (%)
Rapa-lip	120.9 ± 0.38 nm	0.126 ± 0.051	− 12.2 ± 0.4 mV	98.20 ± 0.15

Under a transmission electron microscope, the rapamycin liposomes were uniform spherical or spheroid-like particles without adhesion and coalescence, as shown in Fig. 1G.

### Method validation

#### Coefficient of diurnal variation and recovery

In Table 2, the coefficients of diurnal variation at three different concentrations and recovery values are summarized. The coefficient of diurnal variation for rapamycin was in the range of 2.09–2.92%. The recovery exceeded 75%, thus indicating that the method was accurate and reproducible for the determination of rapamycin in mouse whole blood and lung tissue.

**Table 2:** Summary of Coefficient of diurnal variation and recoveries (n = 3)

	CV (%)	Blood-recovery (%)	PIF-recovery (%)
0.5 µg/mL	2.35	97.50 ± 4.01	79.28 ± 8.30
0.1 µg/mL	2.09	–	–
0.02 µg/mL	2.92	–	–

#### Linearity, LLOQ and LOD

The correlation coefficient (R^2^) for the standard curve was 0.9993 for a concentration of rapamycin ranging from 0.001 to 0.05 μg/mL and 0.9990 for a concentration of rapamycin ranging from 0.05 to 1 μg/mL. All calibration curves were found to be adequate for the analysis of mouse blood and lung samples. The LLOQ of rapamycin was identified to be 0.001 µg/mL, which was sufficient for pharmacokinetic study. The LOD of rapamycin was 0.0001 µg/mL.

### Pharmacokinetic application

**Fig. 3:**
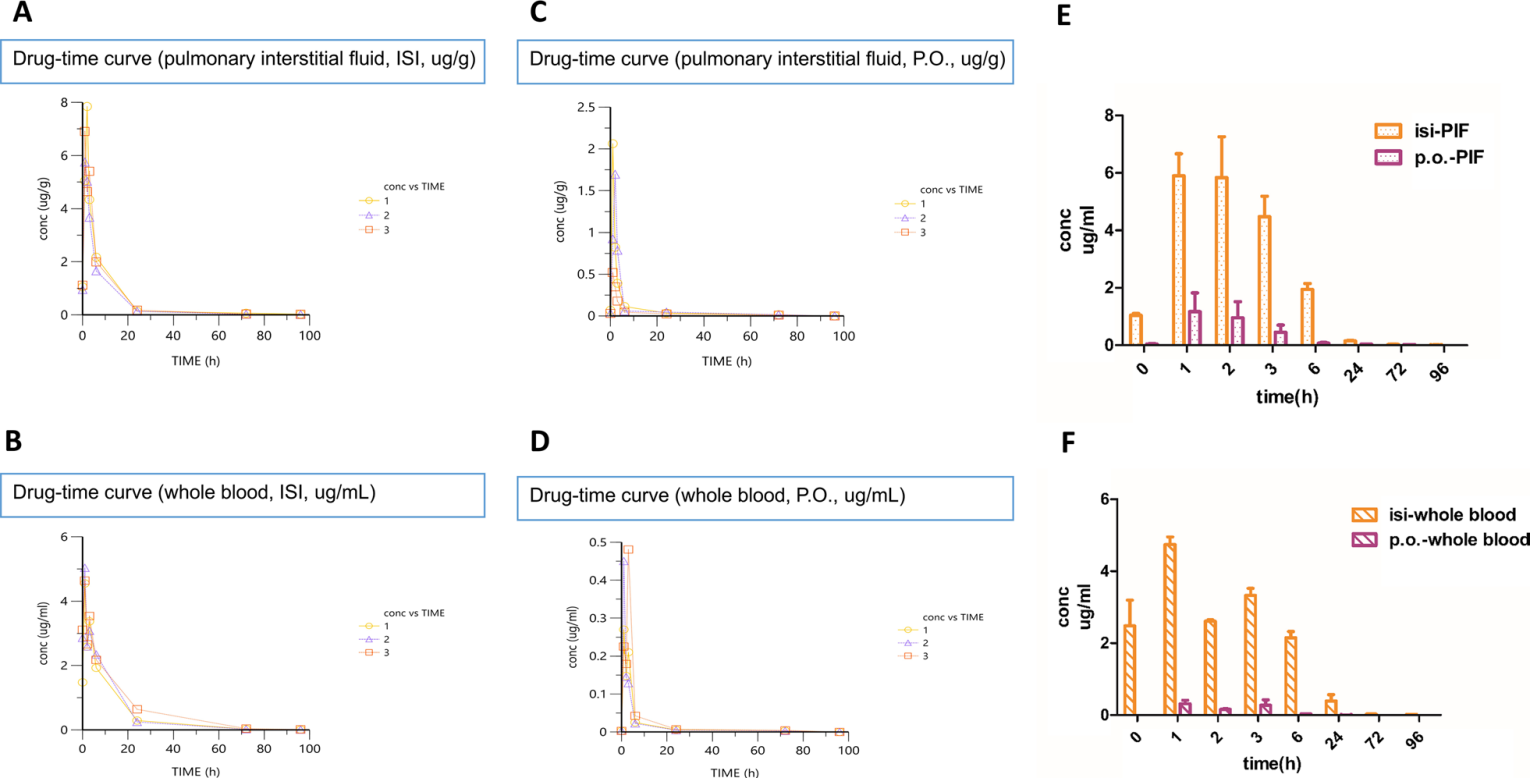
Drug-time curves of whole blood and pulmonary interstitial fluid by P.O. or ISI. **A** Drug-time curve of pulmonary interstitial fluid by ISI. **B** Drug-time curve of whole blood by ISI. **C** Drug-time curve of pulmonary interstitial fluid by P.O. **D** Drug-time curve of whole blood by P.O. **E** Time profile of drug content in pulmonary interstitial fluid after ISI or P.O. administration. **F** Time profile of drug content in whole blood after ISI or P.O. administration

The LC–MS/MS method was applied in a preliminary pharmacokinetic study of rapamycin in mice. The whole blood and pulmonary interstitial fluid concentration–time curves and bar charts of rapamycin after oral (100 mg/kg) or ISI (100 mg/kg) administration of rapamycin are shown in Fig. 3.

Primary pharmacokinetic parameters calculated with noncompartment model analysis are summarized in Table 3. The results demonstrated that the concentrations of rapamycin were clearly higher after ISI administration than oral administration. The F value of rapamycin was determined to be 9.83 ± 4.62 in pulmonary interstitial fluid and 31.70 ± 3.65 in whole blood (F = F_isi_/F_p.o._). On the basis of the greater F with ISI administration, we concluded that the administration method of interstitial injection with nanoparticles achieved better efficacy.

**Table 3:** The pharmacokinetic parameters of rapamycin in mouse whole blood and pulmonary interstitial fluid (PIF) after oral (P.O.) or intervaginal space injection (ISI) administration

	p.o.PIF	isi.PIF	p.o.blood	isi.blood
T_max_ (h)	1.33 ± 0.47	1.33 ± 0.47	1.67 ± 1.15	1.00 ± 0.00
C_max_ (µg/g or µg/mL)	1.43 ± 0.66	6.84 ± 0.86	0.40 ± 0.09	4.74 ± 0.21
AUC_0→72 h_ (h * µg/g or h * µg/mL)	5.30 ± 1.80	47.44 ± 4.51	1.61 ± 0.38	51.48 ± 6.79
AUC_0→∞_ (h * µg/g or h*µg/mL)	5.46 ± 1.84	48.87 ± 4.91	1.69 ± 0.42	52.16 ± 6.98
CL (10^3 ^* g/h/kg or 10^3 ^* ml/h/kg)	19.77 ± 8.37	2.07 ± 0.22	62.45 ± 13.29	1.95 ± 0.24
F (F_isi_/F_p.o._)	9.83 ± 4.62		31.70 ± 3.65	

C_max_, peak concentration; T_max_, time to C_max_; AUC, area under the concentration–time curve; CL, plasma clearance; F, bioavailability

## Discussion

PLAM is a malignant tumor that is characterized by diffuse proliferation of abnormal smooth muscle fibers, and is relatively rare and refractory. Interstitial fluid is the main source of lymph fluid, and the delivery of drugs through the interstitial fluid may be more suitable than traditional blood delivery for such interstitial diseases. With the aim of increasing the amount of drugs reaching the interstitial fluid, we chose ISI as the mode of administration, on the basis of the close connection between PLAM and the interstitium [7, 8].

Since the development of modern medicine, a variety of routes of administration have been achieved, including oral and intravenous injection, but each has drawbacks. For instance, oral administration often has low bioavailability [9, 10], whereas intravenous injection can irritate systemic blood vessels or result in systemic toxicity [11]. The new drug delivery method of ISI combined with nanoparticles was based on the hierarchical porous thick collagen bundle network in human interstitial tissue and driven by the orderly flow of interstitial fluid. The interstitial space, the primary source of lymph and a major fluid compartment in the body, has not been appreciated enough since its discovery. In 1894, Burnett [12] reported a clinical case of a tumor growing in the intervaginal space of the optic nerve, emphasizing that the interstitial space, such as the parenchymal tissue, plays an important role in the occurrence and transformation of the disease. In recent years, the interstitial space, which is not between cells but is a macroscopically visible space within tissues through which interstitial fluid flows around the body, has received increasing attention by researchers. Feng et al. [13] have reported a “green pathway” different from simple diffusion in tissue, explained by the properties of fluid flow within the interstitial space of tissue in the presence of a multiphase porous media structure. Li et al. [14] have used fluorescein, NMR and CT to confirm the existence of long-range transmission pathways originating from the extremities in the human body. Several years later, Benias et al. [15] demonstrated the microstructure of the interstitial space in bile ducts and skin, which is defined by a complex lattice of thick collagen bundles, according to electron microscopy and tissue slices. The fluid in interstitial spaces also have a different transmission direction from blood. Injecting gold nanoparticles through interstitial space channels results in a markedly different tissue distribution and metabolic processes than does intravenous injection [16]. Treatment of diseases including breast cancer [6] and malaria [17] with ISI has also been investigated.

Compared with traditional oral administration, ISI can avoid a series of intestinal adverse reactions caused by oral drug delivery, and can increase the bioavailability of drugs. The bioavailability of oral preparations of rapamycin has been reported to be low, and the degree of oral absorption is greatly affected by the type of food eaten [18]. These aspects must be considered during the administration of rapamycin to achieve steady state drug concentrations. In the intravenous injection method, the dose and volume of the drug are often relatively large, and a systemic reaction can easily occur. ISI is administered by injecting the drug into a specific sheath in the interstitium; the drug is then transported through the interstitial space, bypassing the blood vessels and liver, and reaches the lesion. The administration method of ISI was found to significantly increase rapamycin bioavailability and decrease the drug clearance rate; both aspects are important for regulating the dosage and frequency of medication. After ISI administration, the content of the drug in the pulmonary interstitial fluid and blood of mice significantly increased; this aspect is important for the treatment of PLAM diseases characterized by abnormal proliferation of smooth muscle cells outside blood vessels and lymphatic vessels. Thus, the drug concentration around the lesion was improved and maintained.

The reason of drug dosage form changes to nano-size is also based on the porous structure of the interstitial network, thus minimizing drug diffusion into the blood. Nanosized drugs are more easily recognized by macrophages and transported to lymphoid tissues. ISI is a new type of drug delivery that differs from traditional drug delivery. Notably, drugs with high binding rates to hemoglobin, such as rapamycin, are not suitable for intravenous injection. Here, we provide a new solution for the delivery of such drugs. Furthermore, studies on ISI are underway, and this delivery mode is expected to pave the way to a new research field in the near future.

## Conclusion

In summary, we found that ISI administration of a drug combined with nanoparticles resulted in significantly greater drug content in the tissue than that with traditional oral administration of the same dose. These findings may have great value in improving drug bioavailability, decreasing adverse drug reactions and enabling more accurate blood concentration monitoring of drugs. Furthermore, this new drug administration route may be well suited for the treatment of refractory interstitial diseases such as PLAM.

## Data Availability

Please contact author for data requests.
